# Development of Long Wavelength Light-Absorptive Homopolymers Based on Pentaazaphenalene by Regioselective Oxidative Polymerization

**DOI:** 10.3390/polym13224021

**Published:** 2021-11-20

**Authors:** Hiroyuki Watanabe, Kazuo Tanaka, Yoshiki Chujo

**Affiliations:** Department of Polymer Chemistry, Graduate School of Engineering, Kyoto University, Katsura, Nishikyo-ku, Kyoto 615-8510, Japan; hiroyuki.watanabe4@konicaminolta.com (H.W.); chujo@poly.synchem.kyoto-u.ac.jp (Y.C.)

**Keywords:** near infrared, azaphenalene, homopolymer, narrow band gap, oxidative polymerization

## Abstract

We report the synthesis and absorption properties of homopolymers consisting of 1,3,4,6,9b-pentaazaphenalene (5AP). Oxidative polymerization in the Scholl reaction was accomplished, and various lengths of homopolymers can be isolated. It should be noted that we scarcely observed the generation of structural isomers at the connecting points, which is often observed in this type of reaction. Therefore, we were able to evaluate electronic structures of the synthesized homopolymers. In addition, it was observed that absorption bands were obtained in the longer wavelength region than the monomer. The computer calculation suggests that the highest occupied molecular orbital (HOMO) energy levels could be lowered by electronic interaction through spatially-separated HOMOs of 5AP. Moreover, we can evaluate the extension of the conjugated system through the meta-substituted skeleton and distance dependency of the main-chain conjugation.

## 1. Introduction

Heteroatom-containing π-conjugated polymers have been regarded as an attracting platform for organic electronic materials due to their optoelectronic properties, such as light-absorption properties, luminescent properties, and carrier-transport properties, which are caused from main-chain conjugation and intriguing electronic properties originating from heteroatoms [1,2,3,4,5,6]. Therefore, the construction of unique electronic structures with various elements is still a relevant topic in this research field [7,8,9,10]. Currently, most of heteroatom-containing conjugated polymers have donor-acceptor type structures in the main-chains. In contrast, homopolymers are an important class of conjugated polymers since homopolymers tend to form an electronic interaction among the monomeric unit. From the previous examples on homopolymers, light-absorption properties with large molar extinction coefficients and narrow HOMO-LUMO gaps can often be observed [11,12]. These electronic properties are favorable for presenting not only high carrier-transport ability, but also electric conductivity by doping.

**Scheme 1:**
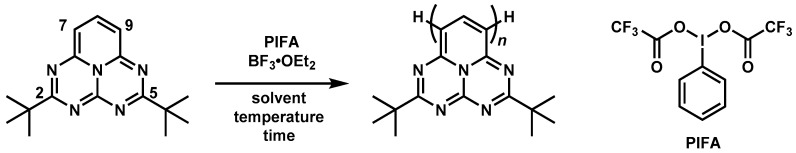
Synthesis of 5AP homopolymers via oxidative polymerization in the Scholl reaction.

We have focused on azaphenalene derivatives as an element-block, which is a minimum functional unit containing heteroatoms [13,14,15,16,17], for constructing conjugated polymeric materials due to their unique electronic structures [18,19,20,21,22,23,24,25]. By extending conjugated systems through polymer main-chains, bathochromic shifts of optical bands are usually observed. Interestingly, significant bathochromic shifts were observed from the donor-acceptor type conjugated polymers involving pentaazaphenalene (5AP, Scheme 1), in which each 5AP unit was connected at the meta position [18]. It was suggested that electronic communication should be extended through the meta-connected 5AP units [18]. Moreover, it was discovered that 5AP forms the π-conjugated systems with the isolated orbitals stemming from the spatially separated frontier molecular orbitals (FMOs) [19,20,21,22,23,24,25]. In the 5AP skeleton, when one of the electronic orbitals of FMOs exists on the skeletal carbon, the node is obtained in another FMO. This character of π-conjugated system is sometimes observed in a certain heterocyclic compound and is far different from those of commodity π-conjugated polymers, where both of the FMOs are delocalized over the main chains of the polymers [12,26,27,28,29,30]. Based on the character of FMOs, we have demonstrated that one of the FMOs can be selectively tuned by the substituent effect, the boron complexation, and connection with other conjugation units [21,22]. As a result, near-infrared-absorbing dyes and deep-red emissive materials were obtained with 5AP [21,22]. In summary, by introducing electron-donating substituents at 7,9-positions, only energy levels of HOMOs can be significantly elevated. Meanwhile, those of LUMOs can be selectively lowered by the introduction of electron-accepting substituents at 2,5-positions. From these data, it was presumed that energy levels might be able to be modulated by the extension of electronic conjugation. To evaluate the validity of this hypothesis, we designed 5AP homopolymers.

Herein, we illustrate the synthesis and electronic properties of homopolymers of 5AP. When the transition-metal catalyzed reactions are not applicable, oxidative polymerization in the Scholl reaction has been utilized. However, we often suffer from the generation of structural isomers at the connecting points due to the intrinsic high reactivity of intermediates. In this study, regioselective polymerization for obtaining homopolymers was observed. In particular, although each monomer unit has steric hindrance, we accomplished the attainment of homopolymers with the single connecting motif. In addition, variable lengths of homopolymers can be isolated with chromatography. From the optical measurements, we evaluated the influence of the extension of conjugated system through the meta-substituted skeleton and distance dependency of the main-chain conjugation on optical properties. We can propose that the heterocyclic compounds which have spatially-separated FMOs are versatile for building robust conjugation systems.

## 2. Results and Discussion

**Figure 1:**
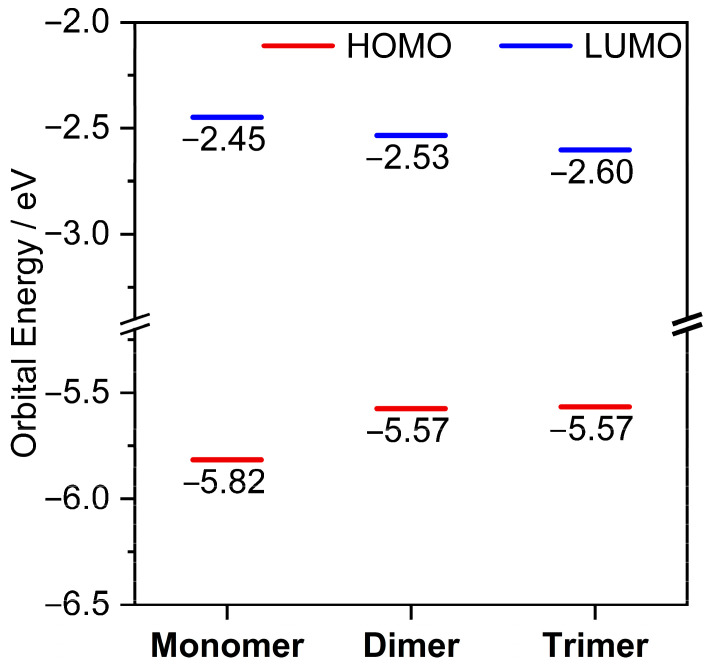
The HOMO and LUMO levels of the monomer, dimer, and trimer calculated at B3LYP/6-31+G(d,p) (monomer) and B3LYP/6-31+G(d,p)//B3LYP/6-31G(d,p) (dimer and trimer) levels of theory.

Initially, the structures and electronic properties were theoretically investigated by density functional theory (DFT) calculations using the Gaussian 16 C.01 package. Optimized structures were estimated with the calculations at the B3LYP/6-31+G(d,p) level of theory for the monomer and the B3LYP/6-31+G(d,p)//B3LYP/6-31G(d,p) level of theory for the dimer and trimer. The calculated orbital energies of the HOMOs and LUMOs are summarized in Figure 1. The extension of the π-conjugated system in the homopolymers was quite different from those in the copolymers. As mentioned above, 5AP has the spatially-separated FMOs [25]. When connected at the 7,9-positions, it is presumable that the delocalized HOMO over the main chain and the isolated LUMO on each 5AP could be obtained since only HOMO is distributed at the connected points. From the calculated data, it was clearly shown that large degrees of the elevation of HOMOs were observed from the dimer and trimer, whereas only a slight lowering was observed from the LUMOs. This result significantly suggests that the selective elevation of HOMO of 5AP can be induced by polymerization, followed by narrow band gaps. Compared to the results from the monomers and copolymers, LUMOs were relatively lowered by the connection at the 7,9-position. Due to steric hindrance between the 5AP units, torsion should be induced (Figure S1). Therefore, the lowering effects of LUMOs could be induced by polymerization.

**Figure 2:**
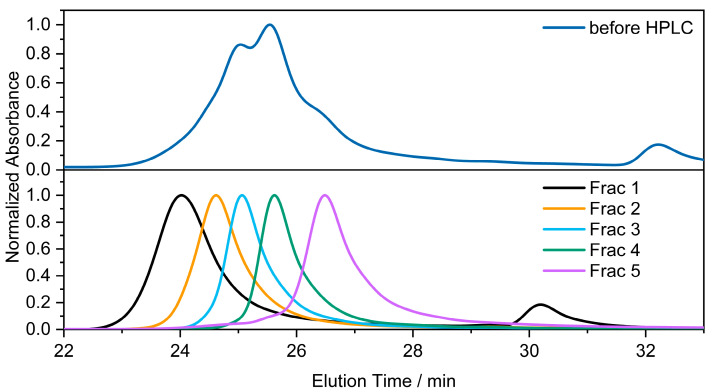
GPC traces of the crude sample (**up**) and the fractionated sample (**down**).

To obtain homopolymers, we preliminary conducted commodity metal-catalyzed reactions, such as the Yamamoto coupling, which is often applied for the attainment of electronic acceptor-consisting homopolymers, with brominated 5AP [31]. However, we obtained insoluble species, and any analyses were not applicable. Therefore, we performed oxidative polymerization in the Scholl reaction using phenyliodine bis(trifluoroacetate) (PIFA) as an oxidizer (Scheme 1) [32,33,34]. Preliminary screening of the solvent and temperature was conducted as described in Table S1, and the crude reaction mixture was analyzed by size-exclusion chromatography (SEC, Figure S2). Consequently, we found the appropriate reaction condition and obtained the polymeric products, according to the SEC profile after the reaction. In order to evaluate the influence of a conjugation length on the electronic properties, we prepared various lengths of polymers by the separation with preparative high-performance liquid chromatography (HPLC). According to the SEC profiles, a series of polymers with variable lengths were obtained (Figure 2). Moreover, we confirmed their molecular weights by matrix-assisted laser desorption ionization time-of-flight mass spectroscopy (MALDI-TOF-MS, Figure S3). From the analyses, it was revealed that the mixture sample was mainly composed of the trimer and tetramer of 5AP. The mass values that increased repeatedly corresponded to the expected monomer unit. The polymer with 10 repeating units was detected from the spectra of the fractionated samples (Figure S4). From the mass values, it was observed that hydrogen atoms were introduced at the chain ends. These data indicate that the undesired byproducts, such as oxidative decomposition products, were not incorporated into polymer chains. From the thermal analyses, a lower decomposition temperature was obtained from the polymer (Figure S5). Torsion through the polymer main-chain might be responsible for instability.

As is often the case with oxidative polymerization, the products contain structural isomers at the connecting points [11]. Therefore, the chemical structures of the polymeric products in Fractions 1–5 were carefully examined by ^1^H NMR spectroscopy (Figure S6). By the comparison of the spectra, it was clearly shown that the products should have the expected structure. The doublet peaks around 6.2 ppm (marked with a black triangle) should be attributable to the peaks of the hydrogen atoms at the 7- (7-H) and 9-positions (9-H). The multiple peaks (marked with a bright green square) were probably assigned as the peaks of the hydrogen atoms at the 8-positions (8-H). It should be noted that they contain a doublet peak originating from the 8-H at the chain-ends. The singlet peak at 7.4 ppm (marked with a red pentagon) was not detected, indicating that coupling reactions selectively occurred at the 7,9-positions of the 5AP unit. Owing to the localization of HOMO, good selectivity in oxidative reactions should be obtained.

**Figure 3:**
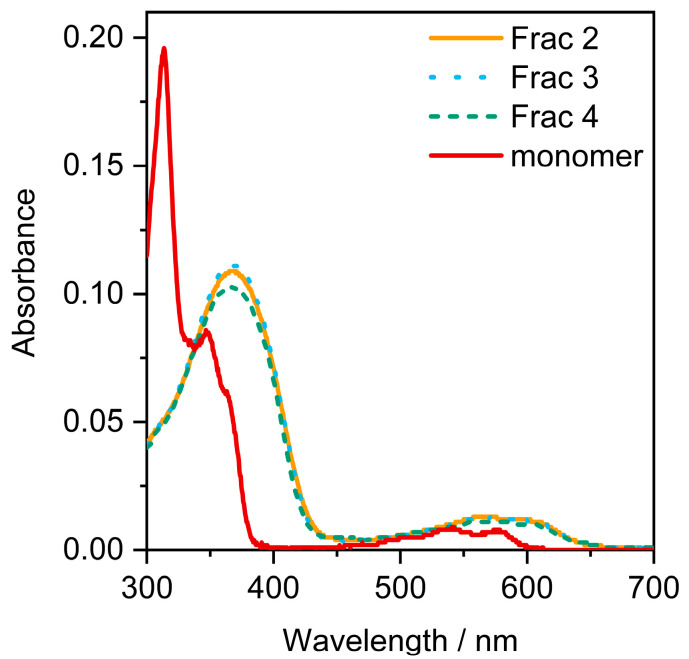
UV-vis absorption spectra of monomer and Fractions 2–4 in the chloroform (1.0 × 10^−5^ M per repeating units).

The extension of the π-conjugated system in the 5AP homopolymers was evaluated by UV-vis absorption spectroscopy. The spectra of Fractions 2, 3, and 4 were measured in the chloroform (Figure 3, 1.0 × 10^−5^ M per repeating units). Fractions 1 and 5 were not used due to the possibility that impurities, such as monomers, could be included. Compared to the spectrum of the monomer, the absorption bands were observed from the polymers in the longer wavelength regions, clearly indicating that electronic conjugation should be elongated through the polymer main-chains. The color of the monomer was purple, meanwhile the homopolymer showed a dark brown color, representing the fact that absorption in the visible region is enhanced by polymerization. It should be noted that the electronic interaction can be obtained through the *meta*-substituted 5AP. The unique π-conjugation can be obtained in homopolymers. Furthermore, corresponding to the calculated data, all of the polymers exhibited similar spectra, suggesting that the effective conjugation length should be around three monomer units. Due to torsion at the connecting points, an electronic interaction should be disturbed. Moreover, significant effects of the chain-ends were hardly observed in the spectra, although the effects from the chain-ends are often critical in the short polymer main-chain. Owing to the hydrogen chain ends, perturbation in the spectra should be suppressed.

## 3. Conclusions

We successfully synthesized homopolymers of 5AP through the oxidative coupling reaction. Traditionally, structural isomers are often generated in this reaction, while side-reactions can be suppressed, probably due to the localized HOMO of 5AP. Owing to the reliable polymer structure, we can analyze their electronic structures. Accordingly, it was shown that electronic conjugation can be obtained through the *meta*-substituted 5APs. As a result, peak shifts to longer wavelength regions were accomplished, indicating that the band gap should be narrowed. Computer calculation results suggest that selective elevation of the energy level of the spatially-separated HOMO should be the origin of peak shifts. These data propose that further narrower band gaps might be obtained by the combination of the modification with electron-accepting groups at 2,5-positions, which can induce critical lowering of LUMO energy levels. Due to the material properties as a polymer, such as film-formability and solubility, these optical properties are favorable for the application of advanced organic optical materials, such as a wavelength convertor for near-infrared light, although the magnitude of absorption should be needed. From our findings, heterocyclic compounds, which often have spatially-separated FMOs, are potential candidates, not only as a monomer for constructing homopolymers, but also as a building block for developing advanced narrow-band gap materials.

## Data Availability

The data presented in this study are available on request from the corresponding author.

## Supplementary Materials

The following are available online at www.mdpi.com/article/10.3390/polym13224021/s1. Figure S1: The LUMO and LUMO+1 of the dimer of 5AP. The bonding and anti-bonding interactions are clearly seen around the 7C-7′C bond in the LUMO and LUMO+1, respectively (red dashed circle). Figure S2: GPC traces of (a) monomer and Entries 1–3 and (b) Entries 4–7. Figure S3: MALDI-TOF-MS spectra of the crude sample (up) and the fractionated samples (down). Figure S4: The enlarged MALDI-TOF-MS spectra of Fraction 4. The *m*/*z* values corresponding to the main isotopic peaks are illustrated. Figure S5: (a) TGA and (b) DSC profiles of 5AP monomer and homopolymer. Figure S6: ^1^H NMR spectra of monomer and the fractionated samples (aromatic region, measured in CD_2_Cl_2_). Table S1. Screening of the reaction conditions for homopolymerization via oxidative coupling.
