# Supplementary material for: Development of Long Wavelength Light-Absorptive Homopolymers Based on Pentaazaphenalene by Regioselective Oxidative Polymerization
Source: Polymers (Basel). 2021 Nov 20;13(22):4021. doi: 10.3390/polym13224021 (PMC8619047; doi:10.3390/polym13224021)
Supplement: Supplementary file 1 [file polymers-13-04021-s001.zip › polymers-1464371-supplementary.pdf]

Supporting Information

Development of Long Wavelength Light-Absorptive Homopolymers Based on Pentaazaphenalene by Regioselective Oxidative Polymerization

Hiroyuki Watanabe, Kazuo Tanaka* and Yoshiki Chujo

Department of Polymer Chemistry, Graduate School of Engineering, Kyoto University

Katsura, Nishikyo-ku, Kyoto 615-8510, Japan

Tel: +81-75-383-2604

Fax: +81-75-383-2605

E-mail: tanaka@poly.synchem.kyoto-u.ac.jp

Experimental Section

General

^{1}H (400 MHz) NMR spectra were recorded on JNM-AL400 spectrometer in CD_2Cl_2 using the residual solvent as an internal standard. UV–vis absorption spectra were recorded on a Shimadzu UV-3600 spectrophotometer. Gel permeation chromatography (GPC) was carried out on a TOSOH 8020 (TSKgel G2000HXL G3000HXL, and G4000HXL columns) instrument using chloroform as an eluent after calibration with standard polystyrene samples. For preparative HPLC, Japan Analytical Industry Co. Ltd., Model LC918R equipped with JAIGEL-1HH and 2HH gel-permeation columns were used. Differential scanning calorimetry (DSC) thermograms were carried out on a SII DSC 6220 instrument by using ~10 mg of exactly weighed samples at heating rate of 10 °C/min. The profiles were recorded from the second monitoring curves after annealing at 100 °C for 10 min, followed by cooling to –100 °C. Thermogravimetric analysis (TGA) was performed on an EXSTAR TG/DTA 6220, Seiko Instrument, Inc., with the heating rate of 10 °C/min up to 500 °C under nitrogen atmosphere. Residual chloroform was removed by keeping in a vacuum oven at 100 °C for 1 h before the TGA measurements.

Theoretical Calculation

DFT calculations were performed using Gaussian 16 C.01 package. All optimized structures were confirmed to be local minima because no imaginary frequency was found. For the dimer and the trimer, single point calculations were performed at these optimized geometries. Molecular orbitals were visualized by Gaussview 6 (isovalue:0.02).

Materials

2,5-Di(*tert*-butyl)-1,3,4,6,9b-pentaaphenalene as a monomer was synthesized according to our previous literature (*J. Org. Chem.* **2019**, *84*, 2768–2778). Phenyliodine bis(trifluoroacetate) and

$BF_3{\cdot}OEt_2$ were purchased from commercial sources and used without further purification. Dehydrated solvents (dichloromethane, chloroform and 1,2-dichloroethane) were purchased and used as received.

Polymerization

Polymerizations were performed using the reaction conditions described in Table S1. The standard protocol is shown here: 2,5-Di(*tert*-butyl)-1,3,4,6,9b-pentaaaphenalene (20 mg, 0.071 mmol) and corresponding amounts of the reagents were dissolved in solvents (1 mL) under nitrogen atmosphere. The mixture was reacted under the reaction conditions as shown in Table S1. For SEC analyses, the mixture sample was directly analyzed after the reaction. In the case of Entry 5, which is the optimized condition, the polymer was obtained as a dark brown solid (18 mg, 89%). For UV–vis absorption spectroscopy and MALDI analyses (dithranol matrix), the crude sample of Entry 5 was purified as follows:

The mixture products (~0.1 M in dehydrated dichloroethane) were diluted by $CHCl_3$ (50 mL). The diluted solution was washed by sat. $NaHCO_3$ aq. (1 × 30 mL) and water (1 × 30 mL). The organic layer was dried over $MgSO_4$, then filtered and solvents were removed *in vacuo*. The sample was purified by preparative HPLC. After the removal of the solvent, the purified and fractionated samples were analyzed.

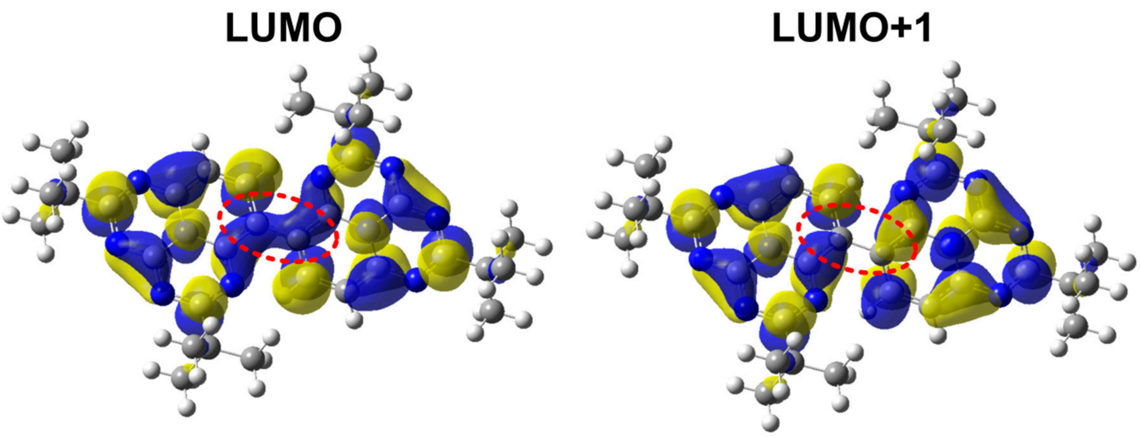

Figure S1. The LUMO and LUMO+1 of the dimer of 5AP. The bonding and anti-bonding interactions are clearly seen around the 7C-7'C bond in the LUMO and LUMO+1, respectively (red dashed circle).

Table S1. Screening of the reaction conditions for homopolymerization via oxidative coupling

Entry	PIFA / eq.	BF_3•OEt_2 / eq.	Solvent	Temp. / °C	Time / h
**1**	2.0	10	CH_2Cl_2	–78	3
**2**	2.0	10	CH_2Cl_2	–78	24
**3**	2.0	10	CH_2Cl_2	rt	18
**4**	2.0	10	$CHCl_3$	50	24
**5**	3.0	3.0	CH_2ClCH_2Cl	70	15
**6**	4.0	4.0	CH_2ClCH_2Cl	70	15
**7**	5.0	5.0	CH_2ClCH_2Cl	70	15

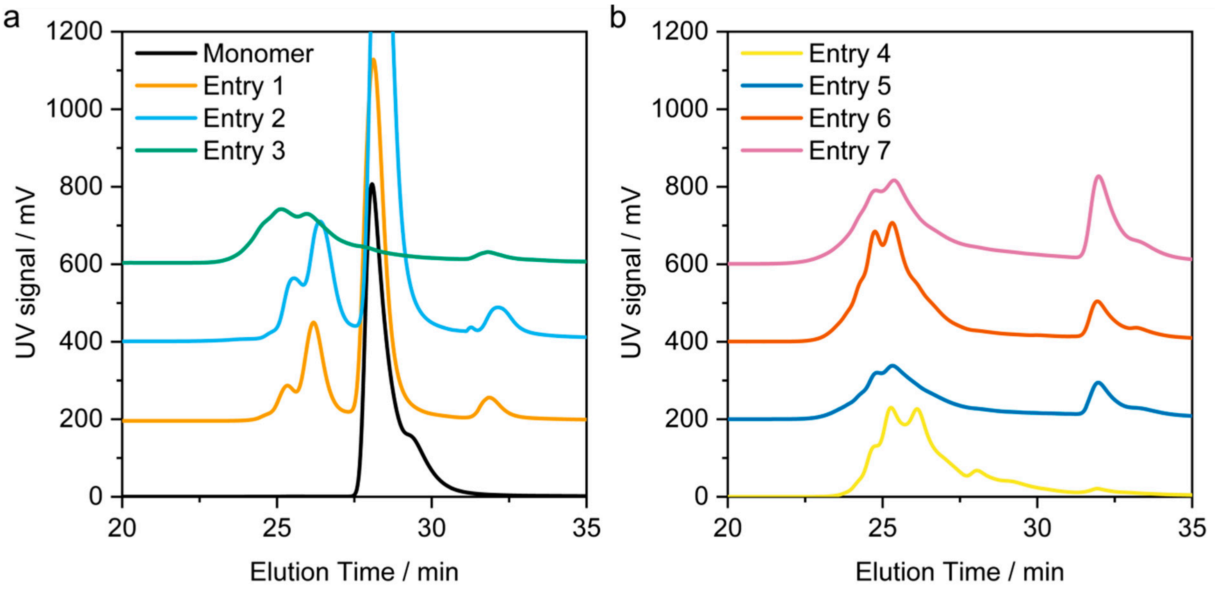

Figure S2. GPC traces of (a) monomer and Entries 1–3 and (b) Entries 4–7.

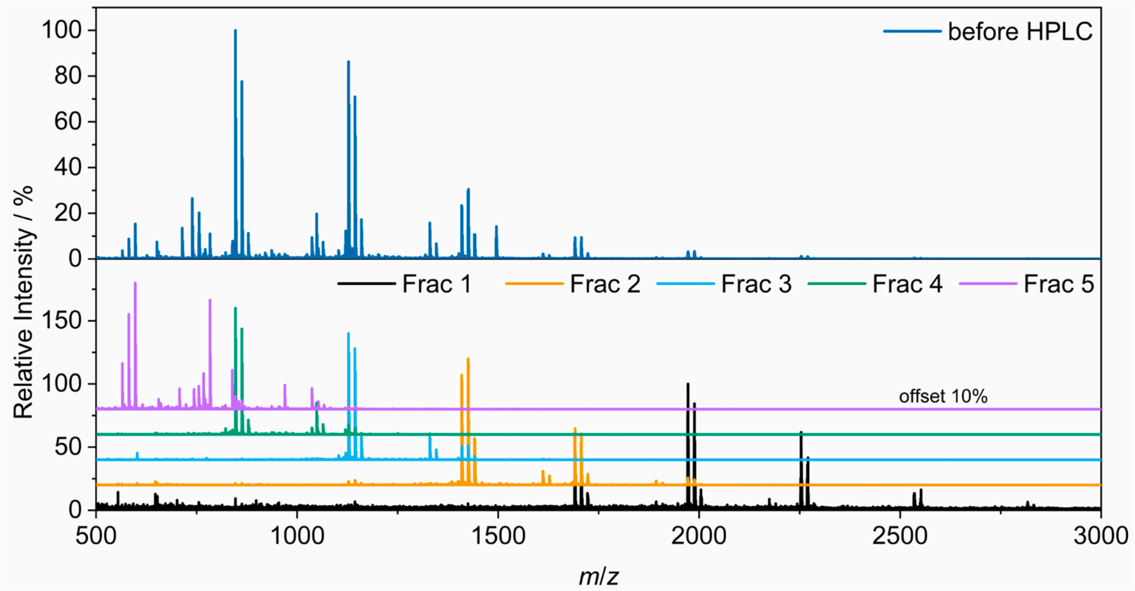

Figure S3. MALDI-TOF-MS spectra of the crude sample (up) and the fractionated samples (down).

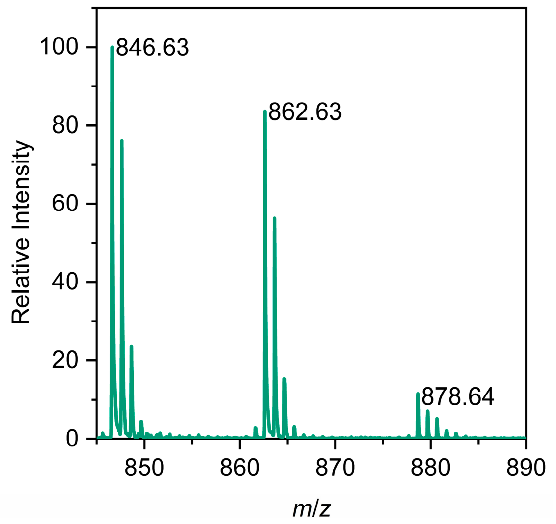

Figure S4. The enlarged MALDI-TOF-MS spectra of Frac 4. *m*/*z* values corresponding to the main isotopic peaks are illustrated.

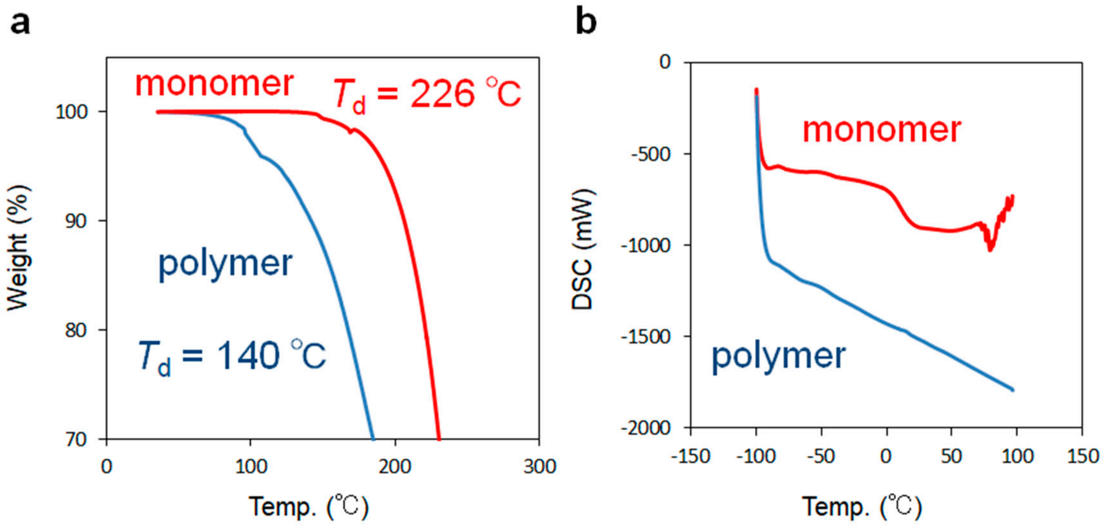

Figure S5. (a) TGA and (b) DSC profiles of 5AP monomer and homopolymer.

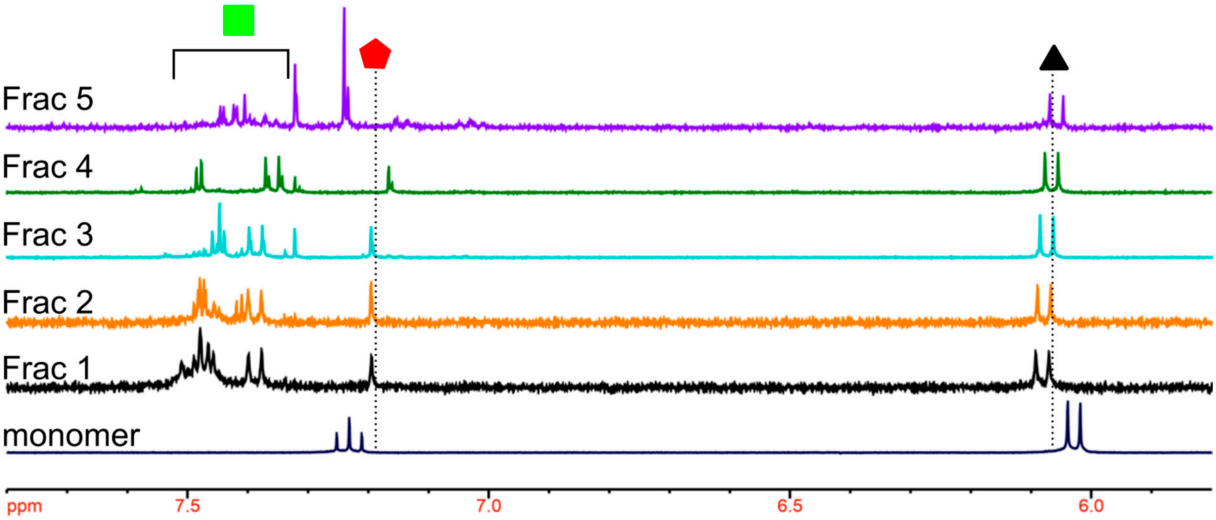

Figure S6. ^{1}H NMR spectra of monomer and the fractionated samples (aromatic region, measured in CD_2Cl_2).
